# OpaR Controls the Metabolism of c-di-GMP in *Vibrio parahaemolyticus*

**DOI:** 10.3389/fmicb.2021.676436

**Published:** 2021-06-07

**Authors:** Yiquan Zhang, Yue Qiu, He Gao, Junfang Sun, Xue Li, Miaomiao Zhang, Xingfan Xue, Wenhui Yang, Bin Ni, Lingfei Hu, Zhe Yin, Renfei Lu, Dongsheng Zhou

**Affiliations:** ^1^Wuxi School of Medicine, Jiangnan University, Wuxi, China; ^2^School of Medicine, Jiangsu University, Zhenjiang, China; ^3^State Key Laboratory of Infectious Disease Prevention and Control, National Institute for Communicable Disease Control and Prevention, Chinese Center for Disease Control and Prevention, Beijing, China; ^4^Department of Clinical Laboratory, Nantong Third Hospital Affiliated to Nantong University, Nantong, China; ^5^State Key Laboratory of Pathogen and Biosecurity, Beijing Institute of Microbiology and Epidemiology, Beijing, China

**Keywords:** *Vibrio parahaemolyticus*, biofilm, quorum sensing, OpaR, c-di-GMP

## Abstract

*Vibrio parahaemolyticus*, the leading cause of seafood-associated gastroenteritis worldwide, has a strong ability to form biofilms on surfaces. Quorum sensing (QS) is a process widely used by bacteria to communicate with each other and control gene expression *via* the secretion and detection of autoinducers. OpaR is the master QS regulator of *V. parahaemolyticus* operating under high cell density (HCD). OpaR regulation of *V. parahaemolyticus* biofilm formation has been reported, but the regulatory mechanisms are still not fully understood. bis-(3′-5′)-cyclic di-GMP (c-di-GMP) is an omnipresent intracellular second messenger that regulates diverse behaviors of bacteria including activation of biofilm formation. In this work, we showed that OpaR repressed biofilm formation and decreased the intracellular concentration of c-di-GMP in *V. parahaemolyticus* RIMD2210633. The OpaR box-like sequences were detected within the regulatory DNA regions of *scrA*, *scrG*, VP0117, VPA0198, VPA1176, VP0699, and VP2979, encoding a group of GGDEF and/or EAL-type proteins. The results of qPCR, LacZ fusion, EMSA, and DNase I footprinting assays demonstrated that OpaR bound to the upstream DNA regions of *scrA*, VP0117, VPA0198, VPA1176, and VP0699 to repress their transcription, whereas it positively and directly regulated the transcription of *scrG* and VP2979. Thus, transcriptional regulation of these genes by OpaR led directly to changes in the intracellular concentration of c-di-GMP. The direct association between QS and c-di-GMP metabolism in *V. parahaemolyticus* RIMD2210633 would be conducive to precise control of gene transcription and bacterial behaviors such as biofilm formation.

## Introduction

*Vibrio parahaemolyticus*, a Gram-negative, rod-shaped, halophilic bacterium, is responsible for the most common seafood-associated gastroenteritis that is often caused by the eating of raw or undercooked seafood (Broberg et al., 2011). *V. parahaemolyticus* has a strong ability to form biofilms on surfaces, which can enhance the survival rate of the bacterial cells in adverse growth conditions as well as the pathogenicity of the bacteria (Yildiz and Visick, 2009; Li et al., 2020). Biofilm formation of *V. parahaemolyticus* requires many specific structures and substances including flagella, type IV pili, capsular polysaccharide, exopolysaccharide, proteins, and extracellular DNA (Enos-Berlage et al., 2005; Shime-Hattori et al., 2006; Yildiz and Visick, 2009; Li et al., 2020). Some regulators such as OxyR (Chung et al., 2016), ToxR (Chen et al., 2018), QsvR (Enos-Berlage et al., 2005), and CpsR (Guvener and McCarter, 2003) and some regulatory processes such as quorum sensing (QS) (Yildiz and Visick, 2009; Ball et al., 2017; Lu et al., 2018) and bis-(3′-5′)-cyclic di-GMP (c-di-GMP) (Yildiz and Visick, 2009) are also required for the regulation of mature biofilm formation by *V. parahaemolyticus*.

Cyclic-di-GMP is a ubiquitous second messenger that controls diverse behaviors of bacteria including motility, virulence, and biofilm formation (Jenal et al., 2017). High concentrations of intracellular c-di-GMP promote the formation of biofilms but inhibit the motility of bacteria (Jenal et al., 2017). c-di-GMP synthesis is catalyzed by diguanylate cyclases (DCs) containing a GGDEF domain with GTP as the substrate, whereas its degradation is catalyzed by phosphodiesterases (PDEs) carrying either an HD-GYP or EAL domain (Jenal et al., 2017). *V. parahaemolyticus* harbors dozens of genes that encode proteins containing GGDEF and/or EAL (or HD-GYP) domains (Makino et al., 2003). The two proteins, ScrG and ScrC, contain both GGDEF and EAL domains, but they act only as PDEs to degrade c-di-GMP in wild-type *V. parahaemolyticus* strains (Boles and McCarter, 2002; Kim and McCarter, 2007; Ferreira et al., 2008; Trimble and McCarter, 2011). ScrC, which is encoded by the *scrABC* operon, acts as a DC to catalyze the synthesis of c-di-GMP in the absence of ScrA and ScrB, whereas it serves on a PDE to degrade c-di-GMP in the presence of ScrA and ScrB (Boles and McCarter, 2002; Ferreira et al., 2008; Trimble and McCarter, 2011). Overexpression of ScrG was sufficient to decrease biofilm formation and intracellular c-di-GMP, whereas removal of the EAL domain converts ScrG to an activator of biofilm formation and c-di-GMP synthesis (Kim and McCarter, 2007). Double gene mutant of *scrG* and *scrC* does not swarm but produces a more highly wrinkled colony than those of the single-gene mutants, suggesting that they have cumulative effects on biofilm formation (Kim and McCarter, 2007). ScrO, a newly described GGDEF-type protein, is encoded by the *scrMNO* operon (Kimbrough et al., 2020). Deleting *scrO* or the entire operon decreases biofilm formation and alters biofilm architecture and matrix production visualized on Congo red (Kimbrough et al., 2020). Three genes, *scrJ* and *scrL* which encode proteins with GGDEF domains, and *lafV* which encodes an EAL domain, were recently found to negatively influence swarming motility in *V. parahaemolyticus* (Kimbrough and McCarter, 2020).

Quorum sensing is a cell-to-cell communication process widely used by bacteria to control gene expression in response to the concentration change of signaling molecules called autoinducers (AIs) in surroundings (Ng and Bassler, 2009). *V. parahaemolyticus* produces three types of AIs: harveyi autoinducer 1 (HAI-1), autoinducer 2 (AI-2), and cholerae autoinducer 1 (CAI-1) (Zhang et al., 2012). At low cell density (LCD), low concentrations of AIs initiate a phosphorylation cascade, which ultimately leads to a high expression level of AphA and a low expression level of OpaR (Lu et al., 2018; Sun et al., 2012). Highly expressed AphA regulates many cellular pathways such as virulence gene expression, motility, and biofilm formation (Lu et al., 2018). By contrast, at high cell density (HCD), high levels of AIs lead to a low expression level of AphA and a high expression level of OpaR (Sun et al., 2012; Zhang et al., 2012). OpaR then regulates transcription of many genes including those associated with virulence, motility, and biofilm formation (Henke and Bassler, 2004; Gode-Potratz and McCarter, 2011; Zhou et al., 2013; Ball et al., 2017; Lu et al., 2019; Zhang et al., 2019). Thus, AphA and OpaR are the master QS regulators in *V. parahaemolyticus* that exert their major roles at LCD and HCD, respectively.

**FIGURE 1:**
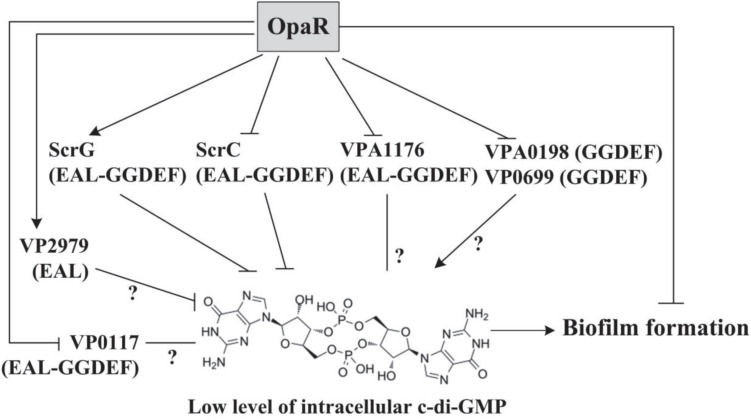
Regulatory model. The arrow lines indicate positive regulation. The T-junctions indicate negative regulation. The question marks indicate that the roles of the protein need to be further investigated. The roles of ScrC and ScrG have been well studied in *V. parahaemolyticus* BB22.

*Vibrio parahaemolyticus* QS regulation of biofilm formation *via* its master regulators AphA and OpaR has been reported (Enos-Berlage and McCarter, 2000; Guvener and McCarter, 2003; Enos-Berlage et al., 2005; Wang et al., 2013), but the regulation mechanisms are still not fully understood to date. In the present work, the results showed that OpaR repressed the biofilm formation by *V. parahaemolyticus* RIMD2210633 at least in part by reducing the intracellular c-di-GMP concentration (Figure 1). The data also showed that OpaR directly regulated the transcription of *scrA*, *scrG*, VP0117, VPA0198, VPA1176, VP0699, and VP2979, encoding a group of proteins with GGDEF and/or EAL domains (Figure 1). Thus, one of the mechanisms of OpaR-dependent biofilm formation in *V. parahaemolyticus* RIMD2210633 is that OpaR can alter the intracellular pool of c-di-GMP.

## Materials and Methods

### Mutation of *opaR* and Complementation of the *opaR* Mutant

*Vibrio parahaemolyticus* RIMD2210633 was used as the wild-type (WT) strain in the current study (Makino et al., 2003). Non-polar *opaR* deletion mutant Δ*opaR* and the complementation of the *opaR* mutant (C-Δ*opaR*) were constructed in our previous studies (Zhang et al., 2012, 2019). Control strains were constructed by transforming the empty pBAD33 vector into WT and Δ*opaR* to counteract the effects of arabinose and chloramphenicol on bacterial growth and physiology (Sun et al., 2014). All the primers used are listed in Table 1.

**TABLE 1:** Oligonucleotide primers used in this study.

Target	Primers (forward/reverse, 5′–3′)
**Protein expression**	
*opaR*	AGCGGGATCCATGGACTCAATTGCAAAGAG/AGCGAAGCTTTTAGTGTTCGCGATTGTAG
**qPCR**	
*scrA*	CACACCACGAACACATTGC/TCAATAGCGTCACGGAATGC
*scrG*	AAGCCGTGGTGGAAGAAGG/GCGTGTTGAGTGCGTTGG
VP0117	GACCACCTCAATAGTTATCTG/TAAGTAGGCTTGGACATCTC
VPA0198	GCATCAGAATCAGCAAGAC/ATGCTTAGCTCCTCTTCTTC
VPA1176	GCCATATTCCAAACTCGTTGTG/TGCGTAAGCCAAGTTGATGAG
VP0699	CTGACACATCGTGATACTTC/TTGATGTTGCAGCTCTTG
VP2979	GCAACTCTCAAGTCATCATC/CAACAACCGTCTTCTATGG
**Primer extension**	
*scrA*	/GACTTTAGTTCCACTTTTTTAGC
*scrG*	/ACTTAGTCAACAGTAAATCGTG
*VP0117*	/GACAATCACACCGATAGCCAG
*VPA1176*	/CACCCCATTCTGTCCAT
**LacZ fusion**	
*scrA*	GCGCGTCGACCATCAAGCCATTTTATGAAAC/GCGCGAATTCGTCGGCTGCGATTAGTCTG
*scrG*	GCGCGTCGACTAGCACGCTTGTGTTGGAC/GCGCGAATTCCAGGGAAATGAAGTAATCATGC
VP0117	GCGCTCTAGACTCACACAACACTTTCTG/GCGCGAATTCAGACAATCACACCGATAG
VPA0198	GCGCGTCGACCTCTGGTTCATTGTCTTG/GCGCGAATTCGTCTTGCTGATTCTGATG
VPA1176	AAAGTCGACTCAGGTACGCTTGCTTCAC/GGGGAATTCCGTTGCTTGGTAGTGGTAATAG
VP0699	GCGCGTCGACGGAGAATACCTAGCAGAG/GCGCGAATTCAGTATCACGATGTGTCAG
VP2979	GCGCTCTAGATTTCTATCCGTTGGCTAC/GCGCGAATTCCTGACTTACATCGTGGAC
**EMSA**	
*scrA*	GAGCGTATATCCAAGTGGTTTG/GTCGGCTGCGATTAGTCTG
*scrG*	TAGCACGCTTGTGTTGGAC/CAGGGAAATGAAGTAATCATGC
VP0117	CTCACACAACACTTTCTG/GCGCGAATTCAGACAATCACACCGATAG
VPA0198	CTCTGGTTCATTGTCTTG/GTCTTGCTGATTCTGATG
VPA1176	TCAGGTACGCTTGCTTCAC/CGTTGCTTGGTAGTGGTAATAG
VP0699	GGAGAATACCTAGCAGAG/AGTATCACGATGTGTCAG
VP2979	TTTCTATCCGTTGGCTAC/CTGACTTACATCGTGGAC
16S rDNA	GACACGGTCCAGACTCCTAC/GGTGCTTCTTCTGTCGCTAAC
**DNase I footprinting**	
*scrA*	TCGCAGTTGAACAAATATGACG/CCATTAAGTGACTTTAGTTCC
*scrG*	TAAATACTATTAGGTAACGCG/CAGGGAAATGAAGTAATCATGC
VP0117	CACACTAAAGGTCACAAGCAAG/AGACAATCACACCGATAG
VPA0198	CTTACCTTGGCGTACCTCTTG/ACTGCTTTCTGGGTGAATAACG
VPA1176	GCCATATTCCAAACTCGTTGTG/TGCGTAAGCCAAGTTGATGAG
VP0699	CCACTCCTCTTACAAGAATGAG/AATATCGGATTCCAAGATTC
VP2979	AGCAGTTCCTCGATTTACCTG/GCTTTTCTAGGCACAAAATGAC
**5′-RACE**	
VPA0198	GCTGATGGCGATGAATGAACACTG/AAAGCGAAAAAGAACAGTGC
	GAACACTGCGTTTGCTGGCTTTGATG/ACTGCTTTCTGGGTGAATAACG

### Growth Conditions

*Vibrio parahaemolyticus* strains were grown as described previously (Zhang et al., 2012; Lu et al., 2019). Briefly, bacterial cells were cultured in 2.5% (w/v) Bacto Heart Infusion (HI) broth (BD Biosciences, San Jose, CA, Untied States) at 37°C with shaking at 200 rpm for 12–14 h. The resultant culture was diluted 40-fold into the phosphate-buffered saline buffer (pH 7.2), and 150 μl of the diluted cells was spread onto an HI plate with a diameter of 5 cm. The bacterial cells were harvested after 6 h of incubation at 37°C to simulate the HCD condition at which OpaR was maximally produced (Lu et al., 2019). When necessary, the medium was supplemented with 100 μg/ml gentamicin, 5 μg/ml chloramphenicol, or 0.1% arabinose.

### Colony Morphology

The colony morphology assay was performed as previously described (Fang et al., 2013). Briefly, *V. parahaemolyticus* strains were grown overnight in HI broth, and 2 μl of each resultant culture was spotted onto the HI agar. The plates were incubated at 37°C for at least 2 days.

### Crystal Violet Staining

The crystal violet (CV) staining was performed as previously described (Fang et al., 2013). Briefly, overnight cell cultures were diluted 50-fold into 5 ml of fresh HI broth and cultured at 37°C to OD_600_ = 1.2–1.4. The resulting cultures were 50-fold diluted respectively into 2 ml of fresh Difco marine (M) broth 2216 (BD Biosciences, Untied States) containing 0.1% arabinose and 5 μg/ml chloramphenicol in glass tubes and then grown at 30°C with shaking at 150 rpm for 48 h. Media with planktonic cells were collected for measurement of OD_600_ values. The surface-attached cells were stained with 0.1% CV. The bound CV was then dissolved with 20% ethanol, and the OD_570_ values were determined. Relative biofilm formation was calculated with the formula: 100 × OD_570_/OD_600_.

### Quantification of c-di-GMP

The bacterial cells were harvested after 6 h of incubation at 37°C to measure the intracellular concentration of c-di-GMP, which was performed exactly as previously described (Gao et al., 2020).

### Quantitative PCR

The quantitative PCR (qPCR) assay was performed as previously described (Gao et al., 2011). Briefly, total RNAs were extracted from the WT and Δ*opaR* strains, respectively. The cDNAs were generated using 1–2 μg of total RNAs and 3 μg of random hexamer primers. The relative mRNA levels of each target gene were determined based on the standard curve for 16S rRNA expression for each RNA preparation. The experiment was performed at least three independent times.

### *LacZ* Fusion and β-Galactosidase Assay

For the *lacZ* fusion assay (Sun et al., 2014; Zhang et al., 2017), the regulatory DNA region of each gene was cloned into the pHRP309 plasmid harboring a promoterless *lacZ* gene and a gentamicin resistance gene (Parales and Harwood, 1993). After verification by DNA sequencing, each recombinant plasmid was transferred to WT and Δ*opaR*, respectively. The resulting transformants were cultured and lysed to measure β-galactosidase activity in the supernatants using a β-Galactosidase Enzyme Assay System (Promega, Untied States). The two-plasmid reporter assay in *Escherichia coli* was exactly performed as previously described (Qiu et al., 2020). Briefly, *E. coli* 100 λpir (Epicentre) bearing the indicated *opaR* expression plasmid pBAD33-*opaR* or the empty pBAD33 plasmid and one of the recombinant *lacZ* plasmids was cultured overnight in Luria–Bertani (LB) broth at 37°C. The overnight cultures were diluted 1:100 into 5 ml of fresh LB broth containing 0.2% arabinose, and incubated at 37°C to the mid-log phase (OD_600_ = 1.2).

### Preparation of 6× His-Tagged OpaR

The coding region of *opaR* was cloned into the pET28a plasmid (Novagen, Madison, WI, Untied States) and then transferred into *E. coli* BL21λDE3 to express His-OpaR (Kleber-Janke and Becker, 2000). Purification of overexpressed His-tagged OpaR (His-OpaR) was performed as previously described (Zhang et al., 2012). The dialyzed His-OpaR was concentrated to a final concentration of 0.3 to 0.6 mg/ml. The purity of the resulting protein was analyzed by SDS-12% PAGE.

### Electrophoretic Mobility Shift Assay

Electrophoretic mobility shift assay (EMSA) was performed exactly as previously described (Zhang et al., 2012). The EMSA result was detected by autoradiography using the Fuji Medical X-ray film (Fuji Photo Film Co., Tokyo, Japan).

### DNase I Footprinting

The DNase I footprinting assay was performed exactly as previously described (Zhang et al., 2012, 2017). The results were detected by autoradiography using the Fuji Medical X-ray film (Fuji Photo Film Co., Tokyo, Japan).

### Primer Extension Assay

The primer extension assay was performed exactly as previously described (Zhang et al., 2012, 2017). The results were detected by autoradiography using the Fuji Medical X-ray film (Fuji Photo Film Co., Tokyo, Japan).

### Experimental Replicates and Statistical Methods

Colony morphology, primer extension, EMSA, and DNase I footprinting assays were the result of two independent experiments with similar (or the same) results. The c-di-GMP quantification, CV staining, LacZ fusion, and qPCR results were of at least three independent bacterial cultures, with values expressed as the means ± standard deviation (SD). Paired Student’s *t-*tests were used to calculate statistical significance with *P* < 0.01 considered significant.

## Results

### OpaR Represses Biofilm Formation of *V. parahaemolyticus*

**FIGURE 2:**
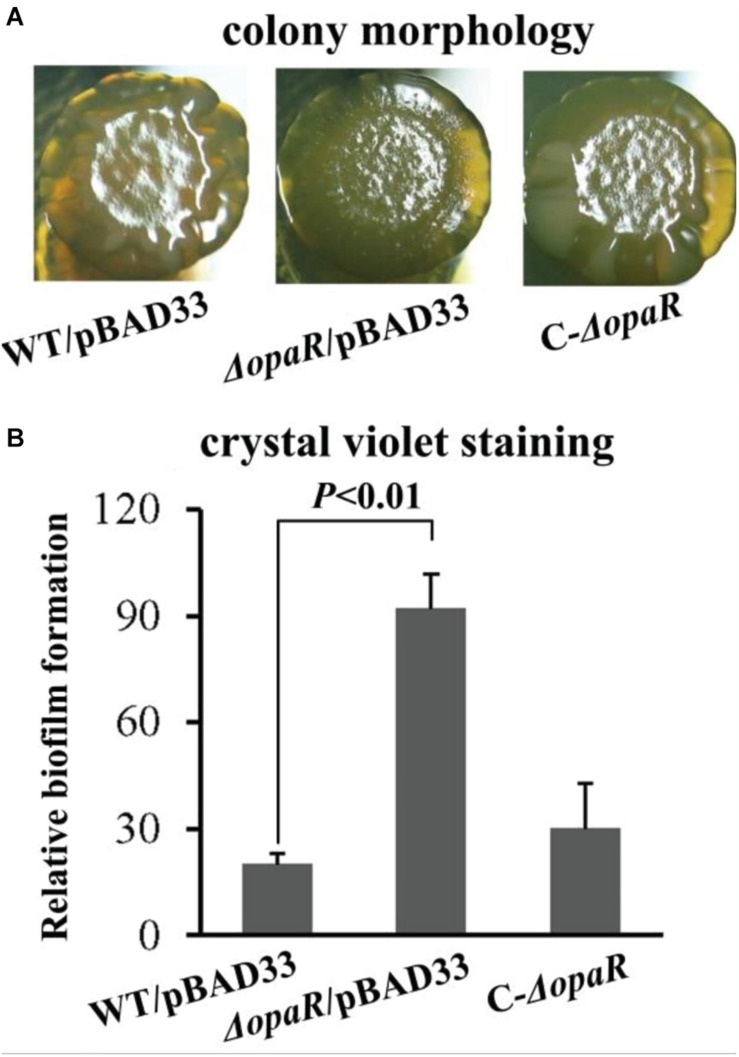
OpaR represses biofilm formation by *V. parahaemolyticus* RIMD2210633. Relative biofilms of *V. parahaemolyticus* RIMD2210633 were measured by rugose colony morphology **(A)** and intensity of crystal violet staining **(B)**. Pictures presented were representative of three independent experiments with three replicates each.

Reports have demonstrated that OpaR controls the expression of numerous genes including those involved in biofilm formation in *V. parahaemolyticus* BB22 (Gode-Potratz and McCarter, 2011; Gode-Potratz et al., 2011; Kernell Burke et al., 2015). However, a study revealed that there are a number of differences in genome sequences between BB22 and RIMD2210633 (Jensen et al., 2013), which may result in different regulatory patterns of OpaR on its target genes between the two strains. In this study, we characterized the biofilm formation regulated by OpaR in *V. parahaemolyticus* RIMD2210633 by comparing biofilm morphology and quantity in WT and Δ*opaR*. As shown in Figure 2A, Δ*opaR*/pBAD33 produced a wrinkled colony, whereas WT/pBAD33 and the complementary opaR mutant (C-Δ*opaR*) formed much smoother colonies than Δ*opaR*/pBAD33. The *in vitro* surface-attached biofilms of *V. parahaemolyticus* strains were subsequently assessed using the CV staining. As shown in Figure 2B, Δ*opaR*/pBAD33 produced a significant increase in normalized CV staining relative to WT/pBAD33 and C-Δ*opaR*, while C-Δ*opaR* gave a restored CV staining. These results suggested that OpaR repressed the biofilm formation by *V. parahaemolyticus* RIMD2210633.

### Deletion of *opaR* Increases the Intracellular c-di-GMP Level

**FIGURE 3:**
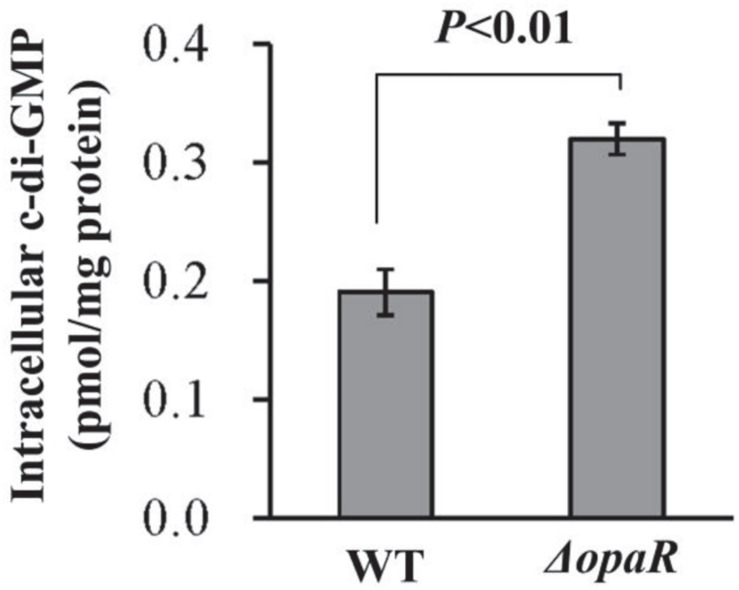
Intracellular c-di-GMP concentrations in *V. parahaemolyticus* RIMD2210633. Bacterial cells were harvested after 6 h of incubation at 37°C on HI plates. The data are expressed as the mean ± SD of at least three independent experiments.

In *Vibrio*, increased c-di-GMP level results in decreased swarming motility and enhanced biofilm formation (Yildiz and Visick, 2009). Thus, increased ability of biofilm formation by Δ*opaR* promoted us to detect whether OpaR regulated c-di-GMP production in *V. parahaemolyticus* RIMD2210633. As shown in Figure 3, a significantly enhanced intracellular c-di-GMP level was observed for Δ*opaR* relative to that for WT, suggesting that OpaR inhibited the production of c-di-GMP in *V. parahaemolyticus* RIMD2210633.

### Predicted OpaR Box-Like Sequences Within the Regulatory Regions of Target Genes

The *V. parahaemolyticus* RIMD2210633 genome harbors at least 50 genes that encode proteins containing GGDEF and/or EAL (or HD-GYP) domains, and their expression or not may influence the intracellular c-di-GMP levels and biofilm formation (Makino et al., 2003). The 500-bp upstream DNA sequences of these genes were downloaded from the RIMD2210633 genome with the online “*retrieve-seq*” program (van Helden, 2003). The DNA-binding box OpaR was then applied to predict the presence of OpaR box-like sequences within these DNA regions using the matrix scan tool (van Helden, 2003; Zhang et al., 2012). The method generated weight scores for each DNA sequence. A higher score represented a higher probability for direct binding of OpaR. When a score of 6 was used as the cutoff value, the OpaR box-like sequences were identified in the regulatory regions of *scrA*, *scrG*, VP0117, VPA0198, VPA1176, VP0699, and VP2979 (Table 2), and thus, these genes were selected as target genes for subsequent gene regulation studies.

**TABLE 2:** Predicted OpaR box-like sequences within target promoters.

Operon	First gene	OpaR box-like sequence				
		Position^&^	Start	End	Sequence	Score
*scrABC*	*scrA*	D	−260	−241	TATAGATAAAACTATTATTA	11.23
	*scrG*	D	−254	−235	TAATGAGTATTCAGTCAATT	10.31
	VP0117	D	−55	−36	TATTAACAGAAATGTCAGTA	12.13
	VPA0198	D	−63	−44	TAATAAGAATTTTAACAATA	10.6
VPA1175-VPA1176	VPA1176	D	−175	−156	TAATGACAATTCAATCATTT	10.76
	VP0699	D	−66	−47	TATAAAGTTTTTTGTTATTA	8.1
	VP2979	D	−232	−213	TATGAATAAAAATGTCATTA	9.5

*^&^“D”indicates the direct sequence. Minus numbers denote the nucleotide positions upstream of the translation start sites of the indicated genes.*

### OpaR Regulates the Transcription of c-di-GMP Metabolism-Related Genes

**FIGURE 4:**
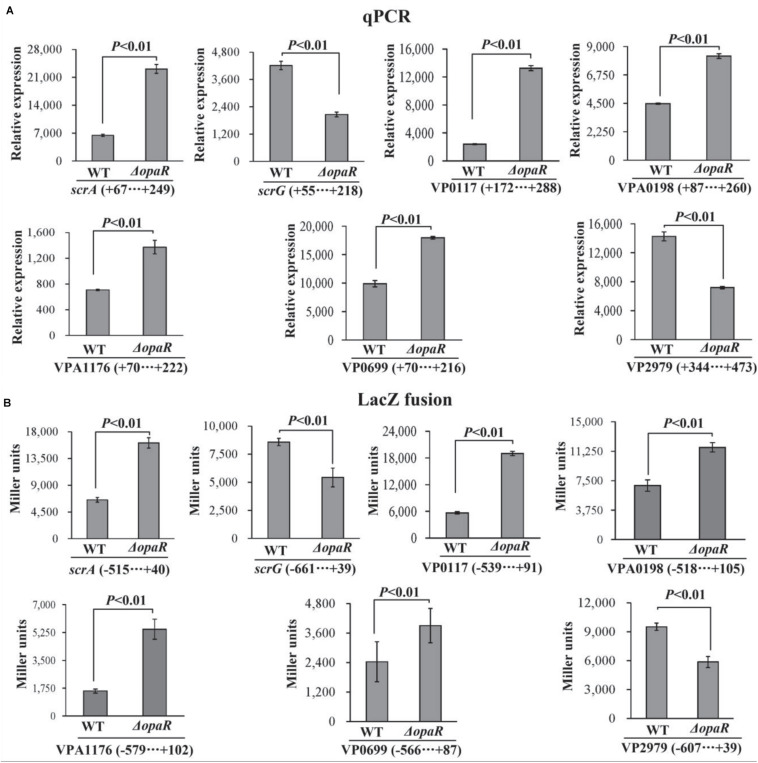
OpaR regulates the transcription of c-di-GMP metabolism-related genes in *V. parahaemolyticus* RIMD2210633. The negative and positive numbers indicate the nucleotide positions upstream and downstream of indicated genes, respectively. Bacterial cells were harvested after 6 h of incubation at 37°C on HI plates, and total RNAs were extracted using TRIzol Reagent. **(A)** qPCR assay was used to determine the relative mRNA levels for each target gene in Δ*opaR* and WT using a standard curve of 16S rRNA expression. **(B)** LacZ fusion. The regulatory DNA region of each target gene was cloned into the pHRP309 plasmid and then transferred into Δ*opaR* and WT to determine the promoter activity in cellular extracts.

The bacterial cells were harvested after 6 h of incubation at 37°C on HI plates, at which the expression level of OpaR was the highest (Lu et al., 2019). The results of qPCR showed that the mRNA levels of *scrA*, VP0117, VPA0198, VPA1176, and VP0699 significantly increased, whereas those of *scrG* and VP2979 significantly decreased, in Δ*opaR* relative to those in WT (Figure 4A). To further investigate whether OpaR has regulatory actions on the promoter activity of each target gene, the upstream DNA region of each target gene was cloned into the pHRP309 vector containing a promoterless *lacZ* gene. The recombinant plasmid for each target gene was then transferred into Δ*opaR* and WT to test β-galactosidase activity in the supernatants. As shown in Figure 4B, the promoter activities of *scrA*, VP0117, VPA0198, VPA1176, and VP0699 were all much higher in Δ*opaR* than those in WT, whereas those of *scrG* and VP2979 were significantly lower in Δ*opaR* relative to those in WT. These results suggested that *V. parahaemolyticus* RIMD2210633 OpaR acted as a negative regulator of *scrA*, VP0117, VPA0198, VPA1176, and VP0699, but it acted as a positive regulator of *scrG* and VP2979.

### OpaR Regulates the Expression of Target Genes in a Heterologous Host

**FIGURE 5:**
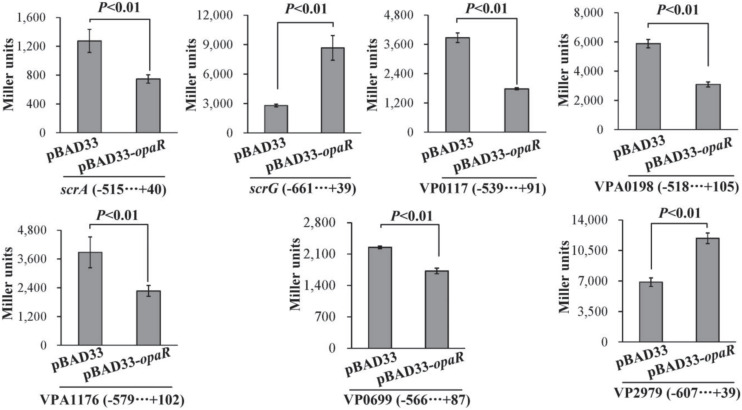
Two-plasmid reporter assay. *E. coli* strains containing the empty pBAD33 plasmid or the indicated *opaR* expression plasmid pBAD33-*opaR* and one of the recombinant *lacZ* plasmids were grown in LB broth containing 0.2% arabinose to mid-log phase (OD_600_ = 1.2), at which point aliquots were collected and assayed for *lacZ* expression using the β-galactosidase assay. The negative and positive numbers indicate the nucleotide positions upstream and downstream of the indicated genes, respectively.

To detect whether OpaR can regulate the expression of target genes in a heterologous host, we expressed OpaR from the pBAD33 arabinose-inducible promoter in the *E. coli* 100 λpir strain, which also contained the recombinant pHRP309 plasmid of one of the target genes (Qiu et al., 2020). As shown in Figure 5, the expression of *opaR* from pBAD33-*opaR* led to a significantly lower promoter activity for each of *scrA*, VP0117, VPA0198, VPA1176, and VP0699 but a significantly higher promoter activity for each of *scrG* and VP2979, when compared to the *E. coli* bearing the empty pBAD33. These results suggested that, in *E. coli*, OpaR bound to the promoter DNA regions of *scrA*, VP0117, VPA0198, VPA1176, and VP0699 to repress their expression, whereas it bound to the promoter DNA regions of *scrG* and VP2979 to activate their transcription.

### His-OpaR Bound to the Regulatory DNA Regions of Target Genes

**FIGURE 6:**
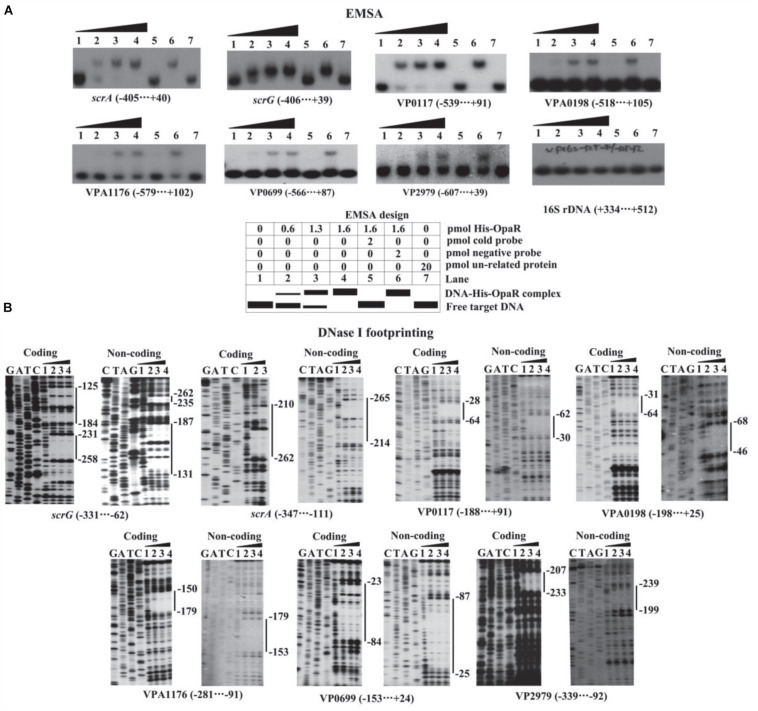
Binding of His-OpaR to the target promoters. The negative and positive numbers indicated the nucleotide positions upstream and downstream of the indicated genes, respectively. **(A)** EMSA. The regulatory DNA fragment of each target gene was incubated with increasing amounts of His-OpaR and then subjected to 6% (w/v) polyacrylamide gel electrophoresis. Schematic representation of the EMSA design was shown below. **(B)** DNase I footprinting assay. Labeled coding or noncoding DNA probes were incubated with increasing amounts of His-OpaR (Lanes 1, 2, 3, and 4 containing 0, 6, 9, and 12 pmol, respectively) and then subjected to DNase I footprinting assay. Lanes G, A, T, and C represent the Sanger sequencing reactions. The protected regions are indicated by the vertical bars on the right side of the image.

The EMSA and DNase I footprinting assays were applied to detect the binding of His-OpaR to the regulatory DNA regions of *scrA*, *scrG*, VP0117, VPA0198, VPA1176, VP0699, and VP2979. The results of EMSA showed that His-OpaR bound to the regulatory DNA fragment of each target gene in a dose-dependent manner but was unable to bind the DNA fragment of 16S rDNA used as the negative control (Figure 6A). As further demonstrated by DNase I footprinting (Figure 6B), His-OpaR protected a single region for each upstream DNA region of *scrA*, VP0117, VPA0198, VPA1176, VP0699, and VP2979, located from 265 to 210 bp, from 64 to 28 bp, from 68 to 31 bp, from 179 to 150 bp, from 87 to 23 bp, and from 239 to 199 bp upstream of the translation start site of the each corresponding gene, respectively. His-OpaR protected two different DNA regions within the regulatory region of *scrG* located from 262 to 231 and from 187 to 125 upstream of the coding region. These results demonstrated that OpaR directly regulated the transcription of *scrA*, *scrG*, VP0117, VPA0198, VPA1176, VP0699, and VP2979 in *V. parahaemolyticus* RIMD2210633.

### Identification of the Transcription Start Sites for the Target Genes

**FIGURE 7:**
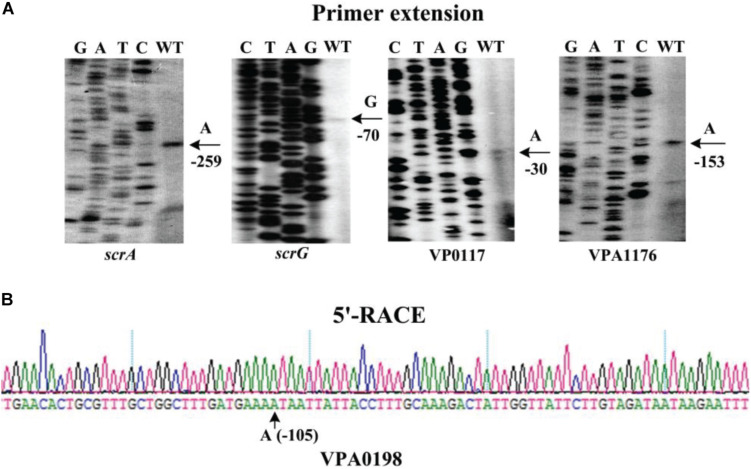
Transcription start sites for target genes. Negative numbers represent nucleotide positions upstream of the translation start site for each target gene. The transcription start sites are marked with arrows and positions. Lanes G, A, T, and C represent the Sanger sequencing reactions. **(A)** Primer extension. An oligonucleotide primer complementary to the mRNA of each target gene was designed. The primer extension products were analyzed with an 8 M urea-6% acrylamide sequencing gel. **(B)** 5′ RACE. The 5′-end of transcripts of target genes were analyzed using the FirstChoice^®^RLM-RACE (Invitrogen, United States) according to the manufacturer’s instructions.

**FIGURE 8:**
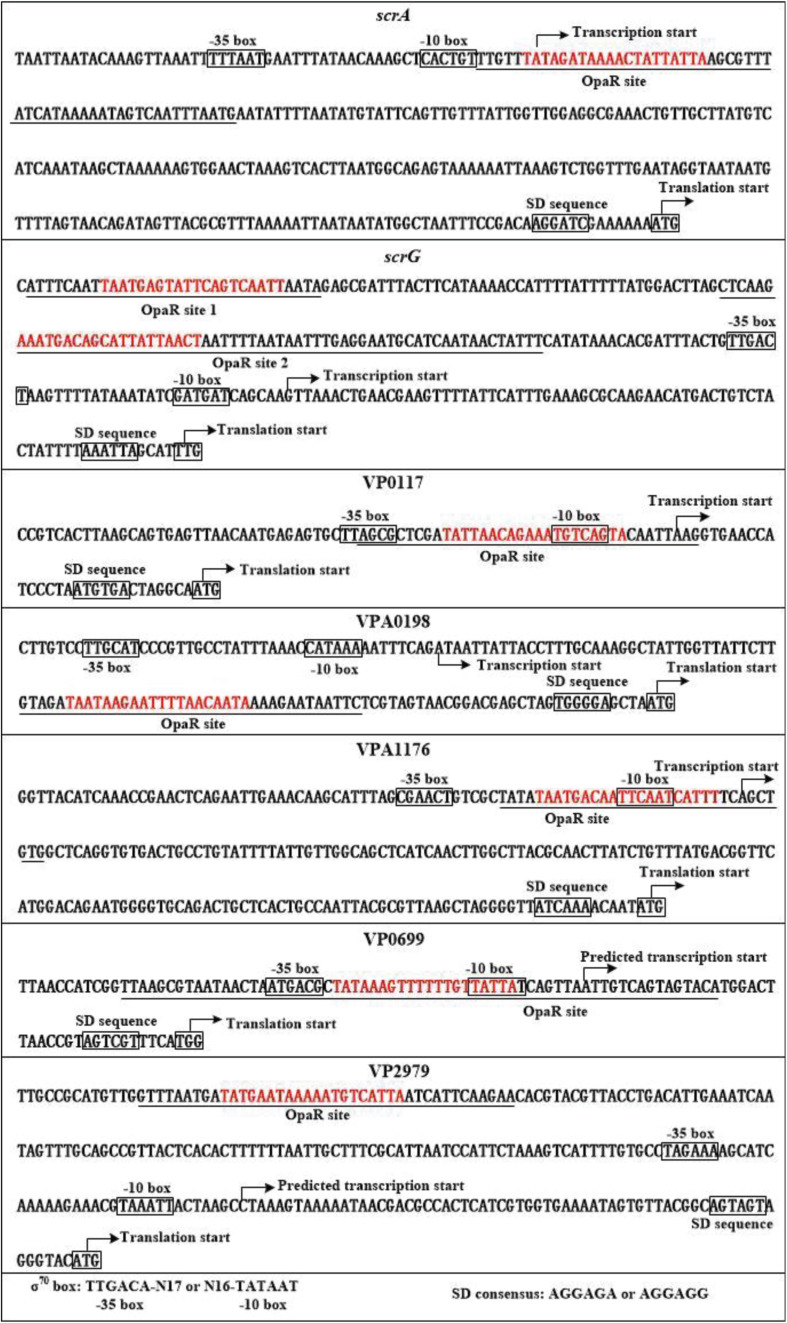
Structural organization of targeted promoters. The DNA sequences were derived from *V. parahaemolyticus* RIMD2210633. Transcription/translation start sites were marked with bent arrows. Shine–Dalgarno (SD) and –10/–35 elements were enclosed in boxes. The OpaR-box like sequences was marked in red. The OpaR sites were underlined with solid lines.

The transcription start sites of each target gene were detected by primer extension assay (Figure 7A). The assay detected a single transcription start site for *scrA*, *scrG*, VP0117, and VPA1176 located at 259, 70, 30, and 68 bp upstream of the corresponding gene, respectively. The putative −10 and −35 elements of each of the transcription start site matched with the consensus prokaryotic sequence (Figure 8). However, the primer extension assay did not detect any transcription start sites for VPA0198, VP0699, and VP2979. Thus, 5′-RACE was further employed to map the transcription start sites for the three genes (Gao et al., 2020). As shown in Figure 7B, the assay only detected one transcription start site for VPA0198 located 105 bp upstream of the translation start site, and its −10 and −35 elements were a good match with the consensus prokaryotic sequence (Figure 8). The assay also did not map any transcription start sites for VP0699 and VP2979. However, a transcription start site located at 37 and 64 bp upstream of the translation start site of VP0699 and VP2979 was predicted separately by using the online tool SoftBerry^1^ (Figure 8). The putative −10 and −35 elements of each start was also a good match with the consensus (Figure 8). Thus, they were considered as the most likely transcription start sites for VP0699 and VP2979.

## Discussion

OpaR was first described to regulate the variation of opaque (OP)–translucent (TR) colony in *V. parahaemolyticus* BB22, which is associated with the production of capsular polysaccharide (McCarter, 1998; Enos-Berlage and McCarter, 2000; Chen et al., 2010). Disruption of *opaR* on the chromosome of an OP strain produced a TR strain, whereas overexpression of *opaR* in a TR strain increased production and converted the strain to an OP type (McCarter, 1998). Approximately 15% of total genes of *V. parahaemolyticus* BB22 were regulated by OpaR, including those involved in capsular polysaccharide synthesis, c-di-GMP metabolism, and type IV pili assemblage (Gode-Potratz and McCarter, 2011; Kernell Burke et al., 2015). Both OP and TR strains formed stable but distinguishable structured biofilms; the OP strain displayed a rapid adherence and then a rapid decline, reaching a plateau at the early stage of the kinetics of biofilm formation, which was higher than that of the TR plateau value (Enos-Berlage et al., 2005). The gene cluster VPA1403–VPA1412 is responsible for the production of exopolysaccharide, a major component of *V. parahaemolyticus* biofilm (Yildiz and Visick, 2009; Chen et al., 2010). OpaR positively regulated the transcription of *cpsA* (VPA1403) and increased the cellular c-di-GMP level (Guvener and McCarter, 2003; Gode-Potratz and McCarter, 2011). Thus, the biofilms formed by *V. parahaemolyticus* BB22 are under the positive control of OpaR.

The data presented here demonstrated that mutation of *opaR* increases the biofilm formation of *V. parahaemolyticus* RIMD2210633 (Figure 2) and the intracellular c-di-GMP concentration (Figure 3). These results are contrary to the previous observations in *V. parahaemolyticus* BB22 (Guvener and McCarter, 2003; Gode-Potratz and McCarter, 2011). We believe that the discrepancies between the presented data and the previous results resulted from the bacterial genetic background. This is because the BB22 genome contains a number of unique genes that the RIMD2210633 genome does not contain, and vice versa (Jensen et al., 2013). Especially, a single missense mutation in *luxO* of *V. parahaemolyticus* BB22 created a quorum-regulatory protein with an altered, constitutively active function, resulting in reduced OpaR activity but a functional QS system, whereas mutations of *luxO* in other *Vibrio* (such as *Vibrio cholerae*) were thought to lock the system in the pathogenic state (Vance et al., 2003; Gode-Potratz and McCarter, 2011). Actually, a similar phenomenon has been also seen in *V. cholerae* that HapR, an OpaR homolog, acts as a repressor of exopolysaccharide production, c-di-GMP synthesis, and biofilm formation (Hammer and Bassler, 2003; Waters et al., 2008).

*Vibrio parahaemolyticus* RIMD2210633 harbors more than 50 genes encoding proteins involved in controlling the intracellular c-di-GMP concentration (Makino et al., 2003). The presented work selected seven genes that might be directly regulated by OpaR as the target genes to investigate the regulatory actions of OpaR on them. The data showed that OpaR bound to the promoter–proximal DNA regions of *scrA*, VP0117, VPA0198, VPA1176, and VP0699 to repress their transcription, whereas it positively and directly regulated the transcription of *scrG* and VP2979. Thus, OpaR transcriptional regulation of seven genes encoding GGDEF and/or EAL-type proteins indeed led directly to changes in the intracellular concentration of c-di-GMP. The QS cascade and c-di-GMP molecule are two important signaling relays controlling gene expression in *V. parahaemolyticus*. The direct association between the master QS regulator OpaR and c-di-GMP metabolism in *V. parahaemolyticus* RIMD2210633 would be conducive to precise control of gene transcription and bacterial behaviors. However, with the exception of *scrA* and *scrG*, functions of the other genes in c-di-GMP metabolism are completely unknown and should be clarified in future studies.

We reconstructed the OpaR-dependent promoter organization of *scrA*, *scrG*, VP0117, VPA0198, VPA1176, VP0699, and VP2979 by collecting data on the transcription start site, core promoter −10 and −35 elements, translation start site, OpaR-binding sites, and Shine–Dalgarno sequences (Figure 8). The OpaR site for each of the regulatory DNA regions of *scrA*, VP0117, VPA1176, and VP0699 overlaps with the −35 and/or −10 elements and/or the transcription start site, and thus, OpaR is thought to repress their transcription by directly interfering with RNA polymerase (RNAP) action. The OpaR-binding site in the promoter–proximal DNA region of VPA0198 locates downstream of the transcriptional start, and thus, the binding of OpaR may block the elongation of RNAP to repress the transcription of VPA0198. The OpaR sites for *scrG* and VP2979 are located upstream of the −35 promoter element, and thus, OpaR positively regulated *scrG* and VP2979 transcription should require class I transcriptional stimulation that depends on the RNAP subunit C-terminal domain to function (Ishihama, 2000).

Taken together, this work reports that the master QS regulator OpaR acts as a repressor of the biofilm formation by *V. parahaemolyticus* RIMD2210633. OpaR regulates the transcription of seven genes encoding a group of GGDEF and/or EAL-type proteins and decreases the intracellular concentration of c-di-GMP. The regulation by OpaR to these genes leads to a reduction in cellular c-di-GMP concentration and then a decrease in biofilm formation.

## Data Availability Statement

The original contributions presented in the study are included in the article/supplementary material, further inquiries can be directed to the corresponding author/s.

## Author Contributions

YZ, DZ, and RL designed, organized and supervised the experiments, interpreted the results, and edited the manuscript. YZ, YQ, HG, JS, XL, MZ, XX, WY, BN, LH, and ZY performed the laboratory experiments. YZ drafted the manuscript. All authors read and approved the final manuscript.

## Conflict of Interest

The authors declare that the research was conducted in the absence of any commercial or financial relationships that could be construed as a potential conflict of interest.

## Footnotes

1 http://linux1.softberry.com/
